# Nanotechnology – a robust tool for fighting the challenges of drug resistance in non-small cell lung cancer

**DOI:** 10.3762/bjnano.14.23

**Published:** 2023-02-22

**Authors:** Filip Gorachinov, Fatima Mraiche, Diala Alhaj Moustafa, Ola Hishari, Yomna Ismail, Jensa Joseph, Maja Simonoska Crcarevska, Marija Glavas Dodov, Nikola Geskovski, Katerina Goracinova

**Affiliations:** 1 Institute of Pharmaceutical Technology, Faculty of Pharmacy, University of Ss. Cyril and Methodius in Skopje, 1000 Skopje, North Macedonia; 2 College of Pharmacy, QU Health, Qatar University, PO Box 2713, Doha, Qatar; 3 Department of Pharmacology, Faculty of Medicine and Dentistry, University of Alberta, T6G 2R3 Edmonton, Canada

**Keywords:** co-delivery nanoparticles, combinatorial therapy, EGFR TKI resistance, non-small cell lung cancer (NSCLC), overcoming and preventing resistance

## Abstract

Genomic and proteomic mutation analysis is the standard of care for selecting candidates for therapies with tyrosine kinase inhibitors against the human epidermal growth factor receptor (EGFR TKI therapies) and further monitoring cancer treatment efficacy and cancer development. Acquired resistance due to various genetic aberrations is an unavoidable problem during EGFR TKI therapy, leading to the rapid exhaustion of standard molecularly targeted therapeutic options against mutant variants. Attacking multiple molecular targets within one or several signaling pathways by co-delivery of multiple agents is a viable strategy for overcoming and preventing resistance to EGFR TKIs. However, because of the difference in pharmacokinetics among agents, combined therapies may not effectively reach their targets. The obstacles regarding the simultaneous co-delivery of therapeutic agents at the site of action can be overcome using nanomedicine as a platform and nanotools as delivery agents. Precision oncology research to identify targetable biomarkers and optimize tumor homing agents, hand in hand with designing multifunctional and multistage nanocarriers that respond to the inherent heterogeneity of the tumors, may resolve the challenges of inadequate tumor localization, improve intracellular internalization, and bring advantages over conventional nanocarriers.

## Introduction

Among the malignant diseases, lung cancer takes the lead in mortality. Also, it is the second most frequently diagnosed cancer (11.4% of the total cases), surpassed only by female breast cancer (11.7%) [1–3]. According to the WHO International Agency for Research on Cancer in 2020 (GLOBOCAN database), around 1.8 million new lung mortalities were recorded worldwide for both genders, representing 18% of all cancer deaths [4]. There are two main classes of lung cancer based on histological appearance, namely small-cell lung cancer (SCLC), which is highly aggressive, and non-small cell lung cancer (NSCLC), which is more prevalent (85% of all diagnosed lung cancer cases) [5]. NSCLC can be further categorized into histologically different subtypes. that is, adenocarcinomas (45%), squamous cell carcinoma (23%), and large cell carcinoma (3%), leaving approximately 28% for all other subtypes and making adenocarcinomas the most prevalent among the subtypes. Recently, based on the progress in cancer genomics, a new classification based on the clinical, histological, radiological, and molecular subtypes of lung adenocarcinoma has been proposed as a result of a joined effort of the International Association for the Study of Lung Cancer (IASLC), the American Thoracic Society (ATS), and the European Respiratory Society (ERS), with the intention of improving diagnostic, prognostic, and therapeutic approaches for different subtypes of lung cancers [6–7]. Moreover, advances in histological, genomic, and proteomic studies of cancer have had a significant impact on the discovery of novel therapies based on specific histological types and molecular signatures of cancer. Molecularly targeted therapies that have been developed for a subgroup of non-small cell lung cancer (NSCLC) with endothelial growth factor receptor (EGFR) activating mutations firmly underlined the importance of an improved classification of lung cancer into specific subtypes that qualify for specialized therapeutic strategies. Tyrosine kinase inhibitors (TKIs) have demonstrated enhanced efficacy and reduced toxicity in EGFR-sensitive patients compared to classical chemotherapy treatments because of their ability to target specific molecular abnormalities associated with NSCLC cells [8–13].

Unlike traditional chemotherapy, which interferes with cell division and kills rapidly dividing cells, molecularly targeted therapy is directed towards somatic genome mutations. Along with the well-established EGFR, Kirsten rat sarcoma viral oncogene homolog (KRAS) oncogene mutations and concurrent anaplastic lymphoma kinase (ALK) and proto-oncogene tyrosine-protein kinase (ROS1) rearrangements, other gene mutations in the context of NSCLC tumorigenesis biomarkers and targets for new clinical therapies include fusions of echinoderm microtubule-associated protein-like 4 and anaplastic lymphoma kinase (EML4-ALK) and mutations of human epidermal growth factor receptor 2 (HER2), phosphatidylinositol 3-kinase (PIK3CA), protein kinase B (AKT), v-raf murine sarcoma viral oncogene homolog B1 (BRAF), mitogen-activated protein kinase 1 (MAP2K1), and mesenchymal-epithelial transition factor (MET). An improved understanding of EGFR driver mutations leads the way to the establishment of personalized clinical therapy based on genomic and proteomic testing, which is becoming a standard of care for patients with advanced NSCLC [13–15].

This review article briefly covers some of the advances in therapeutic protocols based on the novel discoveries in molecular profiles and mutational diagnostics of NSCLC, which harbor activating and resistance EGFR mutations along with corresponding genetic alterations leading to drug resistance. Further, an emphasis will be put on the developmental challenges of targeted nanomedicines for the co-delivery of therapeutic agents to lung tumors. Finally, current approaches in literature used to design nanotools loaded with logical combinations of different drugs and inhibitors of various oncogenic pathways to fight NSCLC resistance are covered.

## Review

### EGFR mutations and current problems in NSCLC treatment

The main reasons behind the limited success of TKI monotherapy in the suppression of lung cancer growth for an extended period are tumor heterogeneity, key signaling pathway alteration, and activation of alternate signaling, which effectively rescue the main inhibited pathway. The oncogenic significance of EGFR and the weak response to TKIs have been the focus of clinical interest for more than a decade, motivating the research community to look deeper into relevant explanations for therapeutic failure and suggest smart solutions for overcoming resistance.

There are five selective EGFR TKIs approved for the treatment of EGFR-mutated NSCLC, namely, gefitinib (GEF) and erlotinib (ERL) (first-generation reversible EGFR TKIs), afatinib (AF) and dacomitinib (DAC) (second-generation irreversible EGFR TKIs), and osimertinib (OS) (third-generation irreversible EGFR TKI). All of these drugs act as therapies of choice for NSCLC with EGFR-activating mutations [16–19].

First-generation EGFR TKIs are well-established molecularly driven therapies for lung cancer harboring specific types of activating EGFR mutations involved in the development of NSCLC (classical sensitive EGFR mutations, i.e., deletions in exon 19 and the single-point substitution mutation L858R in exon 21) [8–9]. Not all tumors with activating EGFR mutations will respond to EGFR TKI treatment. A subgroup of around 20–30% of patients harboring an activating mutation is intrinsically resistant to TKIs and shows weak clinical response, including those with wild-type EGFR NSCLC cancer. The establishment of clinical criteria for intrinsic resistance is still ongoing, and considerable efforts are made toward the estimation of the efficacy and optimal sequence of administration of different EGFR TKIs across TKI-sensitive patients with common and uncommon EGFR mutations [20]. The type of EGFR mutation influences the effectiveness of gefitinib and erlotinib across NSCLC tumors. For example, gefitinib is more efficient in treating NSCLC harboring the L858R mutation than in NSCLC expressing the G719S mutation [21]. Further, an approved treatment with EGFR TKIs for NSCLC with defined but uncommon mutations such as G719X, S768I, and L861Q is the second-generation TKI afatinib. Lastly, osimertinib is a third-generation TKI that is approved for EGFR T790M positive NSCLC with acquired resistance to first- and second-generation drugs [12,22–23]. Similar to traditional cytotoxic agents, acquired resistance to TKIs and early relapse are still significant limitations of this therapeutic approach. Following the initial pronounced response, after only 9 to 14 months around 50% of patients develop resistance to therapy with first- and second-generation TKIs as a result of T790M secondary mutation in exon 20 of EGFR [24]. Acquired resistance to TKIs is unavoidable and has already been documented even for the third generation of TKIs (target-dependent and target-independent molecular mechanisms of resistance to TKIs are presented in Table 1). Target-independent acquired resistance driven by cMET amplification after EGFR TKI treatment may be treated with crizotinib, a dual inhibitor of ALK and cMET, and brigatinib (multi-kinase inhibitor of EGFR, ALK, FLT3, and other kinases). This therapy shows effectiveness against mutant variants of EGFR and ALK that are resistant to common types of EGFR and ALK inhibitors [25].

**Table 1:** Molecular mechanisms of resistance to EGFR TKIs.

Target-dependent		

**Secondary exon 20 mutation:** EGFR T790 (40–55% of EGFR resistance cases) and EGFR amplification **Secondary mutations with low occurrence**: D761Y (exon 19), L747S (exon 19), and T854A (exon 21)	**Tertiary mutation**: EGFR C797S; L798I Other tertiary mutations: WZ4002, L718Q, and L844V	
Secondary (T790M gatekeeper residue) and tertiary kinase domain (C797S) resistance mutation in the targeted kinase reduces drug affinity or prevents access of the TKI to the active site and reduces its efficacy. Resistance to gefitinib and erlotinib is evident when the T790 mutation is present, and the C797S mutation induces resistance to osimertinib.		

Target independent		

Bypass resistance (bypass the EGFR blockade) **MET gene amplification:** Amplified MET causes phosphorylation of ERBB3. Even when phosphorylation of EGFR is inhibited by an EGFR TKI, activation of the PI3K/AKT pathway is maintained through ERBB3 or the ERBB3/ERBB2 duet. **HGF overexpression:** HGF induces activation of the PI3K/AKT pathway through MET; this activation is independent of ERBB3 or EGFR). **HER2 amplification:** HER2 forms heterodimers with EGFR to activate downstream signaling.	Downstream signaling mutations in BRAF and PI3K, KRAS, PTEN loss, NF-1 loss, and CRKL amplification, MAPK pathway activation by mutated KRAS or MEK (less frequent events)	Phenotypic alterations epithelial-mesenchymal transition (ЕМТ), or small cell histologic transformation

However, only a limited number of mutations are covered by clinical therapy. In addition, some novel therapeutic approaches against resistant tumors have failed due to the heterogeneity of the progression of genetic alteration and the resulting complexity of resistance mechanisms [23]. Enormous efforts have been made in finding a way forward from this standstill, and evidence has been derived that no single drug can treat the broad spectrum of molecular alterations in NSCLC. Considering the fact that multiple mechanisms are involved in the reactivation of the EGFR signaling pathway, targeting multiple constituents within the EGFR cascade or targeting parallel pathways to prevent cross talk between multiple growth factor receptors have emerged as valid approaches that could be used to tackle cancer resistance and maximize the efficacy of EGFR inhibition. Presently, a combinatorial therapeutic strategy is believed to be a rational approach to combat the complexity of resistance and continuous cancer mutations. Co-delivery of TK inhibitors with anticancer drugs, immunotherapy, or gene-specific therapeutics to disrupt key resistance pathways, reactivate p53-mediated apoptosis, or inhibit cellular drug efflux are only a few examples of strategies used to fight cancer resistance mechanisms successfully [23–24]. In addition, co-delivery of anticancer therapy using surface-engineered nanoparticles for tumor targeting may alleviate some of the unwanted effects on off-site targets and increase the therapeutic concentration at the site of action as well as efficacy and safety of the current therapy for lung cancer treatment. Co-delivery of combined therapeutic agents at the right time and at the right place using smart nanotools to exert a simultaneous effect on multiple signaling pathways, leading to the avoidance or combating of resistance as well as the prevention of side effects, is the theoretical rationale behind the use of designed nanoparticles (NPs) [26–28].

### Advances in the therapeutic approaches used for overcoming NSCLC resistance

Combinatorial treatments are designed with the goal of exerting additive or synergistic inhibitory effects on the proliferation and survival mechanisms on which the cancer cells are heavily dependent. The efficacy of existing small molecules in synergistic combinations for relevant genetic mutations in resistant cancers has been evaluated in many research and clinical studies, with promising results in some types of mutant lung cancers. A plethora of multimodal treatments for the co-administration of: (i) conventional cytotoxic agents with signaling pathway inhibitor/s, (ii) inhibitors of two or more signaling pathways within a signaling network, (iii) inhibitors of multiple targets within a single pathway exerting synergistic effects, and (iv) cytotoxic or molecular targeting agents with small interfering RNA (siRNA) for silencing the mutating genes at protein and messenger RNA (mRNA) level, have made their way to clinical therapy or are under evaluation for their efficacy and safety in many research studies and several clinical trials. The synergistic effects of combination therapy depend on the status and the type of genetic alteration; as such, the most potent will be the one showing synergistic or additive effects on oncogene pathways essential for cell survival.

#### Conventional cytotoxic agents with signaling pathway inhibitor/s

**Figure 1:**
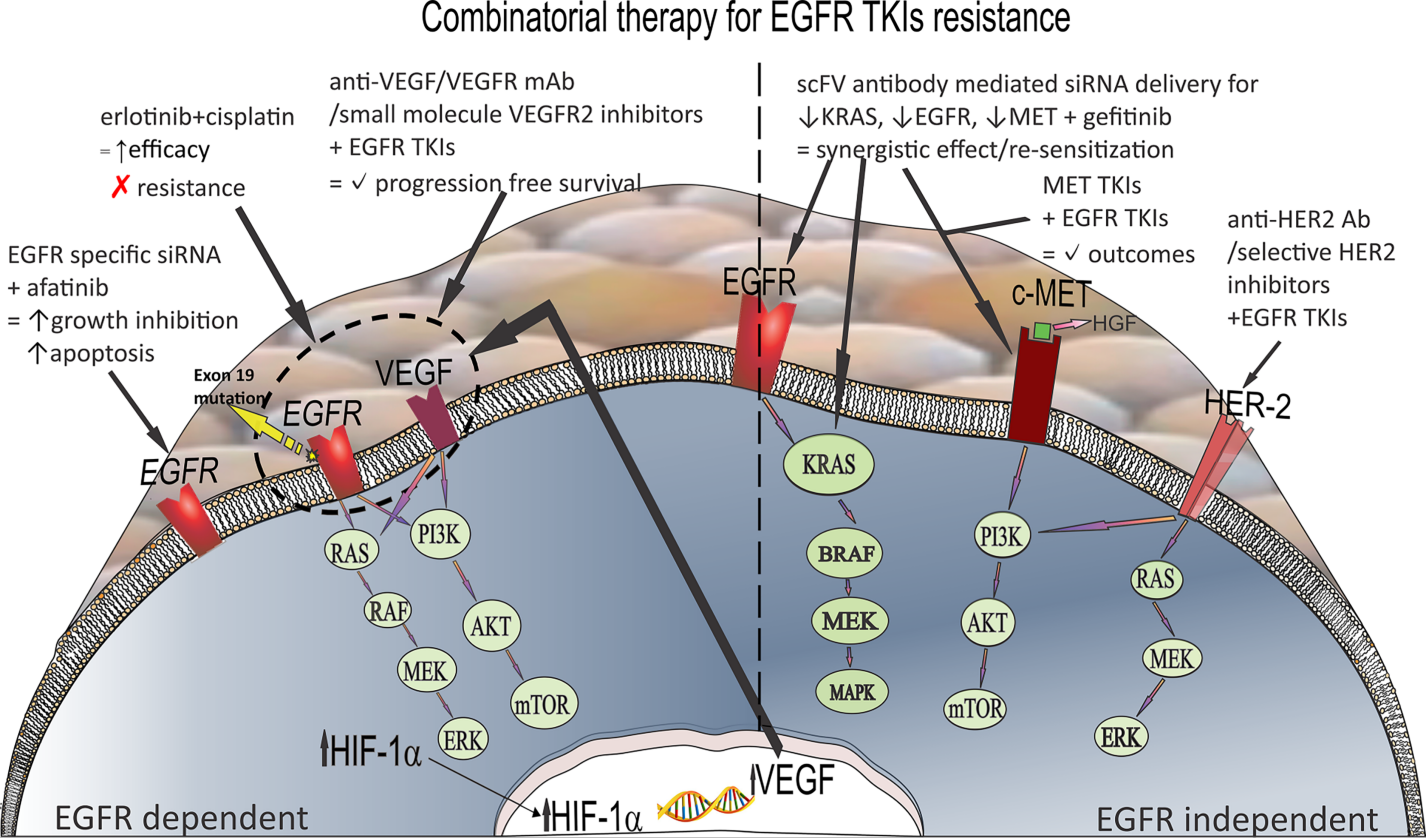
Examples of combinatorial therapy for (left) EGFR-dependent and (right) EGFR-independent TKI resistance. The upward-pointing black arrows indicate upregulation/increase, while the downward-pointing black arrows indicate inhibition/decrease. The black tick marks indicate a beneficial effect. The red x mark indicates a delayed or prevented event.

EGFR signal transduction pathways can be roughly divided into a pro-survival arm with the PI3K-mTOR-AKT cascade and a proliferative arm with the Ras-Raf-Mek-Erk cascade. Enhanced kinase activity on mutated EGFR with exon 19 deletion is associated with upregulated c-MYC levels through the Ras-Raf-Erk pathway, promoting angiogenesis via hypoxia-inducible factor 1α (HIF-1α) and vascular endothelial growth factor (VEGF) signaling [29–31]. According to preclinical data, a combination therapy consisting of erlotinib and cisplatin targets angiogenesis and manifests synergistic and additive antitumor activity via downregulation of the c-MYC–HIF-1α–VEGF signaling pathway in mutated NSCLC with exon 19 deletions (Figure 1) [32–34]. Several randomized clinical studies have also reported on the increased effectiveness of combined chemotherapy/EGFR TKI treatments in patients with NSCLC bearing an EGFR mutation. Therefore, to prevent or delay the emergence of acquired resistance to EGFR TKIs, adding carboplatin and pemetrexed to gefitinib is recommended as a first-line option in patients with EGFR-mutated tumors [35–40].

#### Inhibitors of two or more signaling pathways within a signaling network

EGFR and VEGF share common downstream signaling, although they may function independently during oncogenesis. Increased VEGF levels, which have been confirmed in cancers with acquired resistance, lead to the preservation of tumor growth when the tumor is under attack from EGFR TKIs. Because of the interplay of mechanisms, combining EGFR TKIs and VEGF inhibitors seems to be a rational approach to combat tumor resistance and increase the efficacy of anti-tumor therapy [41–45]. Several trials that compared the efficacy and safety of EGFR TKIs from the first, second or third generation combined with anti-angiogenic therapy, such as small-molecule inhibitors of VEGFR-2 (vandetanib, nintedanib, axitinib, and cediranib) or anti-VEGF/VEGFR monoclonal antibodies (ramucirumab, which is specific for the VEGFR-2 extracellular domain, or bevacizumab, a VEGF-A inhibitor), have been conducted to evaluate the efficacy of combination therapy in mutated NSCLC (Figure 1). Results from preclinical and clinical trials point to substantially improved progression-free survival in patients with EGFR-mutant NSCLC receiving combined EGFR TKIs and angiogenic therapy. However, this combined therapeutic approach has been characterized by an increased incidence of adverse reactions of grades 3–5 [41,46–53]. Nanomedicines can be used as tools for improved localization of combination therapy at the site of action, improving the outcome and decreasing the adverse effects.

#### Inhibitors of multiple targets within a single pathway exerting synergistic effects

Amplification, overexpression, and mutation of MET and HER2 are heavily involved in EGFR TKI resistance development, and the cross talk of these receptors is a way to avoid TK inhibition in many cancers (Figure 1). Hence, these oncogenic drivers are legitimate therapeutic targets in NSCLC with off-target mechanisms of acquired resistance to first- and second-generation TKIs. Various MET TKIs, among them crizotinib and the more selective savolitinib, tepotinib, and capmatinib, may be good candidates for EGFR TKI/MET TKI combination therapy (Figure 1). This approach shows improved clinical outcomes over chemotherapy or MET TKI monotherapy in patients with advanced EGFR-mutant NSCLC who acquired MET amplification or MET overexpression during EGFR TKI treatment [54–62].

Further, substantial evidence for the efficacy of EGFR TKIs combined with HER2-targeted therapy in patients with developed EGFR TKI resistance due to HER2 amplification can be found in the literature [63–65]. Patients with HER2 gene mutations showing resistance to EGFR TKIs may be sensitive to novel, more selective HER2 inhibitors (poziotinib and pyrotinib), HER2 targeting agents such as anti-HER2 antibodies, and small-molecule EGFR tyrosine kinase inhibitor dual therapy (Figure 1). The administration of target-specific antibody–drug conjugates (cytotoxic chemotherapy) has also been characterized by positive responses regardless of HER2-specific mutation sites. In oncogene-addicted cancers (HER2-mutant cancer, oncogene-driven EGFR-positive, ALK-positive, or RET-positive NSCLC), current studies do not encourage the use of immune checkpoint inhibitors, with the exception of KRAS-mutated cancers [61,64–65].

Despite the encouraging results of antibody–drug conjugates, acquired resistance to these agents might eventually develop following the initial positive response. Various mechanisms of acquired resistance in patients with HER2-positive locally advanced breast cancer or metastatic breast cancer involving factors crucial for their mechanism of action are reported across the literature [66–67]. This suggests that such acquired resistance might also become a common problem in advanced NSCLC treatment [68]. Some of the potential factors of resistance, such as poor internalization, defective intracellular trafficking of the HER2 antibody–drug conjugates, masking of the HER2 epitope, high rate of recycling, and the effect of upregulated drug efflux pumps, may be resolved by novel nanomedicines designed to interact with the tumor cells in a variety of ways with the goal of overcoming the limitations of the conjugates [68–69].

#### Cytotoxic or molecular targeting agents with siRNA

Targeting homologous mRNA sequences in cells and knockdown of receptors involved in cell survival and proliferation using RNA interference downregulates receptor protein expression, inhibits cell growth, and induces apoptosis. The effect obtained by siRNA is not influenced by the receptor alteration status and significantly decreases the gene's oncogenic potential. Chen et al. compared the efficacy of TKIs in NSCLC cells harboring different mutations with combined therapy consisting of TKIs (gefitinib, erlotinib, and afatinib) and EGFR-specific siRNA. The authors noted that combined therapy with the potent irreversible EGFR/HER TKI afatinib and EGFR-specific siRNA resulted in enhanced growth inhibition and apoptosis due to the inhibitory effect of the EGFR-specific siRNA on the overall EGFR oncogenic activity, including the downstream TKI resistance mutations (Figure 1) [70]. Lu et al. tested the efficacy of combined siRNA treatment with gefitinib in several NSCLC cell lines (A549, H1975, and H1993). The A549 cell line carried wild-type EGFR and KRAS mutations, H1975 cells expressed L858R/T790M EGFR, and H1993 cells harbored MET amplification. Therefore, three types of siRNA were used as EGFR–scFv–arginine nonamer peptide fusion protein complexes, namely siRNA for KRAS, EGFR, and MET gene expression silencing (Figure 1). The synergistic effect of gefitinib and scFv antibody-mediated siRNA delivery for silencing the expression of resistance-related genes was evidenced by a significant reduction in cell growth and increased rate of apoptosis compared to the cells treated with siRNA only. Furthermore, considering that these cell lines are EGFR-positive TKI-resistant NSCLC cells, a synergistic effect of gefitinib and siRNA may be regarded as proof of restored sensitivity of EGFR-positive NSCLC to gefitinib as a result of silencing the expression of resistance-related genes [71]. Therapy consisting of EGFR siRNA combined with EGFR TKIs and anti-EGFR monoclonal antibodies can additively enhance growth factor inhibition in vitro, maintaining its biological efficacy in cells and xenograft models with different mutation statuses [70,72–74].

#### Further advances in the multimodal combination therapy approach

KRAS proteins operate as guanosine diphosphate (GDP)/guanosine triphosphate (GTP) molecular switches in response to activated transmembrane receptors such as EGFR. The KRAS mutation occurs at a frequency of around 30% in NSCLC, with the KRAS p.G_12_C mutation being the most frequent variant. Mutated KRAS cannot return to its inactive GDP form, which triggers EGFR-independent activation of several downstream effectors [75–77]. The binding of KRAS-GTP to several effectors, among them PIK3K and RAF kinases, triggers activation of downstream AKT and mTOR (PIK3K), which regulates apoptosis, metabolism, and translation, as well as MEK and ERK signaling (RAF kinases), which influences cell cycle progression and proliferation [78]. Therefore, it is expected that KRAS-mutated tumors would not respond to EGFR TKIs. Patients with KRAS-mutant NSCLC can benefit from direct KRAS inhibitors, such as sotorasib, which lock KRAS in its inactive GDP-bound form. However, a heterogeneous resistance pattern during KRASG_12_C inhibitor treatment has been noticed after an initial positive response [78–80]. Co-targeting of upstream signaling (suppression of receptor tyrosine kinases) and downstream signal inhibition by targeting the RAF-MEK-ERK signaling cascade are tested in clinical studies as relevant approaches to delay resistance and improve KRASG_12_C inhibitor efficacy [81–82]. Clinical data from CodeBreak 100/101 revealed promising efficacy with long-lasting anti-tumor effects when a programmed cell death protein 1 (PD-1) antibody was administered alongside a KRASG_12_C inhibitor, suggesting that PD-1 inhibition produces a synergistic effect with sotorasib and enhances CD8-positive T-cell infiltration, which causes an inhibition of tumor growth [83–87]. In addition, there is substantial evidence that the co-delivery of siRNA that shows specific binding to mRNA of the most commonly occurring KRAS missense mutations together with a chemical EGFR inhibitor may efficiently reduce mutant KRAS-induced effects and may contribute to overcoming resistance in the treatment of NSCLC [72,88–89].

Many of the obstacles to the co-delivery of combined therapies can be resolved by nanomedicines as tools for the targeted delivery of high concentrations of anticancer drugs at their site of action. Although designated as molecularly targeted therapies, the targeting of receptors by EGFR TKIs and other receptor inhibitors is not absolute. Once the EGFR TKIs are absorbed from the gastrointestinal tract and distributed in the body, they interact with EGFR signaling pathways of many normal cells influencing their proliferation, differentiation, migration, and apoptosis. This leads to side effects, including rash, erythema, diarrhea, gastrointestinal perforations, ocular lesions, and hematological disorders. Using nanomedicines as a vehicle for the administration of TKIs may alleviate the aforementioned problems of conventional administration and (i) improve their pharmacokinetic profile, (ii) increase tumor targeting potential and localization at the tumor site, (iii) decrease the exposure of healthy tissues to the drug, (iv) minimize off-site targets and side effects, (v) even bypass, reduce, or reverse the multidrug resistance mechanisms, and/or (vi) overcome acquired resistance and sensitize mutant NSCLC cells to EGFR TKIs through the synergistic action of combined therapy against various multiple anti-tumor targets [20,22,90].

### Surface-engineered nanoparticles for lung tumor targeting and co-delivery of combinatorial therapy

Simultaneous delivery of combinatorial inhibitors with the goal of targeting multiple constituents within a single pathway or different oncogenic pathways in therapeutic concentrations at the tumor site, preferentially in the tumor cell, is essential for the efficacy of the therapy. Adequate concentrations might not be achieved with conventional dosage forms mainly due to the poor localization of the free drug molecules at the site of action and the differences in bioavailability and pharmacokinetic parameters. Notably, the failure of delivery at the right time and at the right place contributes to severe systemic toxicities and ineffectiveness. Successful translation of scientific knowledge of the mechanisms of resistance combined with nanotechnology as a tool for targeted delivery may bring improvements in the efficacy of anticancer drugs and may aid in elucidating the beneficial synergistic combinations regarding lung cancer subtype treatment. Nanomedicines have the potential for (i) multivalent targeting and co-delivery of agents to endothelial cells, tumor microenvironment, and tumor cells, (ii) delivering large payloads of active substances with different physicochemical properties, such as small-molecular drugs and siRNA, to the site of action, and (iii) limiting drug resistance [91]. Nanotherapy can change the landscape of clinical lung cancer treatment by mitigating the risk of therapeutic failure due to the non-coordinated co-delivery of therapeutic agents and off-target side effects. However, despite substantial progress, a precise control of the in vivo trajectories of the nanosystems is still beyond our reach. Some of the promising approaches and design considerations in the engineering of tumor-homing nanoparticles will be discussed below with an emphasis on increased lung tumor tissue localization.

#### Current approaches for overcoming biological barriers and improved drug targeting

Looking back at almost half a century of research on drug targeting, experimental evidence shows that efficient tumor localization and intracellular delivery may still be very challenging. However, an improved understanding of the mechanisms involved in angiogenesis, tumor–stroma interactions, molecular heterogeneity between cancers, genetic and epigenetic alterations, and cancer marker expression has not only improved current therapeutic plans for cancer patients but has had an impact on the design approaches of the nanotools for cancer imaging and anticancer drug delivery. In recent years, new platforms to enhance the low tumor targeting capacity of nanomedicines using biomimetic targeting motifs, multifunctional and multistage nanomicelles and polymer nanoparticles, and nanostructured lipid nanocarriers, combined with precision oncology research to identify additional targetable biomarkers, have emerged. Some have been applied in the co-delivery of clinically relevant combinations of molecularly targeted drugs, chemotherapeutic agents, and siRNA.

Historically, the most promising first-generation, passive targeting, stealth polymer NPs for anticancer drug/gene delivery are hydrophobic core–hydrophilic shell NPs including (i) self-assembled kinetically stable amphiphilic block copolymer core–shell NPs, (ii) polymer–polypeptide hybrid core–shell NPs, and (iii) polymer–lipid hybrid core–shell NPs additionally decorated with ligands for overexpressed receptors on cancer cells [92]. Traditionally selected overexpressed cancer cell surface markers for the active targeting of NPs include ανβ3 integrin, aminopeptidase N (CD13), lymphocyte homing receptor (CD44), programmed death ligand-1 (CD274), folate receptor protein, nucleolin receptor, epidermal growth factor receptor (EGFR), vascular endothelial growth factor receptor (VEGFR), human epidermal growth factor receptor 2 (HER2), luteinizing hormone-releasing hormone (LHRH) receptor, and somatostatin receptors (SSTRs) [93].

Due to the complexity of the problem of specific targeting, secondary to the many different types of barriers in the body, incorporating several functionalities to address diverse barriers might improve the targeting efficacy of nanomedicines. Various solutions have been proposed to improve mononuclear phagocytic system (MPS) evasion, extravasation at the tumor site, and diffusion through the dense collagen matrix of the solid tumors. Biomimetic, multifunctional, and multistage targeted nanoscale delivery systems with improved potential for intratumor and intracellular localization, as well as sub-cellular targeting, capable of tackling several body barriers and tumor heterogeneity more efficiently, have been designed to address the problems of efficient targeting [94].

**Multifunctional stimuli-responsive nanosized drug delivery carriers:** Mixed-layer and multilayered nanocarriers with bioresponsive and cleavable layers, possessing different functional properties for improving the enhanced permeability and retention (EPR) effect, diffusion in the tumor microenvironment, cellular internalization and subcellular targeting, were synthesized by click coupling reactions or arranged by self-assembly and co-assembly of block copolymers. These carriers may challenge different barriers after bioresponsive cleavage of the above functionalities. One recently published example involves a micellar structure composed of a polycaprolactone (PCL) core, a mixed poly(2-dimethylamino)ethyl methacrylate/poly(ethylene oxide) (PDMAEMA^(TPP+)^/PEO) middle layer, and a PEO corona. The system was self-assembled using poly(ethylene oxide)-poly(ε-caprolactone)-*b*-poly(ethylene oxide) (PEO_113_-*b*-PCL_70_-*b*-PEO_113_) and poly(2-(dimethylamino)ethyl methacrylate)-*b*-poly(ε-caprolactone)-*b*-poly(2-(dimethylamino)ethyl methacrylate (PDMAEMA_20_^(TPP+)^-*b*-PCL_70_-*b*-PDMAEMA_20_^(TPP+)^) block copolymers. PEO-PCL blocks were linked using acetal groups to enable the cleavage of the PEO blocks from the NP surface in the acidic environment of a tumor and the lysosomes, exposing the PDMAEMA layer decorated with triphenylphosphonium (TPP) ligand to the environment. The TPP lipophilic cation is characterized by a large hydrophobic surface area, which facilitates its permeation throughout phospholipid bilayers, lysosomal escape due to the proton sponge effect, and further accumulation within mitochondria [95]. Barthel et al. developed mixed-layer ABC triblock terpolymer mixed PEO shell nanomicelles of a size below 30 nm, based on poly(ethylene oxide)-*b*-poly(allyl glycidyl ether)-*b*-poly(*tert*-butyl glycidyl ether) (PEO-*b*-PAGE-*b*-PtBGE). The PAGE segment can be subsequently modified using thiolene chemistry to introduce positive charges [−NH_2_ (cysteamine, ENT)], negative charges [−COOH (3-mercaptopropionic acid, ECT)], and active targeting ligands [thiogalactose residues (EGT)] for fine-tuning the charges in the shell in different biological environments either for higher uptake or reduced toxicity. In brief, the ABC triblock terpolymers comprised identical A and C segments. Yet, different functionalities in the middle PAGE block (B) are directly correlated to the combination and the number of functionalities and, therefore, easily adjusted to optimize the systems for different target sites, which is especially promising for nucleic acid delivery [96]. One example of multifunctional, multilayer, bioresponsive lipid polymer nanoparticles with a cleavable layer as a vessel for the co-delivery of erlotinib and bevacizumab was recently published by Pang and co-workers. Clinical studies point to the serious toxicity of conventional application, which might be mitigated with nanotools for co-delivery of therapy for the dual inhibition of VEGF and EGFR pathways. The designed nanocarrier was composed of a polycaprolactone core with bevacizumab and erlotinib, coated with a phospholipid layer, with anchors composed of hyaluronic acid–adipic acid hydralazide–poly(ethylene glycol) (HA-ADH-PEG). Hyaluronic acid-decorated lipid polymer hybrid nanoparticles (LPH NPs) specifically target overexpressed CD44 at the NSCLC cells. In the acidic tumor environment, the pH-responsive linker between HA and PEG is hydrolyzed, leading to the cleavage of the HA layer. This, in turn, decreases the NP size and enables faster tumor diffusion, improved internalization, and drug release at the site of action. These nanocarriers exhibited a high degree of tumor homing, low toxicity, and efficient tumor inhibition in vitro and in a tumor mouse model [97]. An additional example of multilayered cleavable pH-responsive nanoparticles for KRAS mutated cancer is described in Table 2.

**Table 2:** Examples of various nanoparticles for the co-delivery of combinatorial therapy for resistant lung tumor treatment.

Type	Co-delivered agents	Description and outcome	Ref.

polymer NPs (PLGA-PEI^a^)	paclitaxel (PTX) + Stat3 siRNA	increased sensitivity of NSCLC cells to paclitaxel due to the silencing of STAT3	[98]
polymer NPs (MPEG-PLA^b^)	ERL + cilengitide (Cilen; integrin αvβ3 inhibitor)	Cilen reversed EGFR resistance to ERL by inhibition of integrin αvβ3, the activator of galectin-3/KRAS/RalB/TBK1/NF-κB	[99]
polymer NPs (PEG-PLA^c^)	ERL + fedratinib (FDTN; JAK2 inhibitor)	re-sensitization of EGFR resistant cancer to ERL due to suppression of JAK2/STAT3 and disruption of EGFR/JAK2/STAT3 axis	[100]
pH-responsive polymer NPs (DOX-PEI+Bcl2 siRNA^d^)	doxorubicin (DOX) + Bcl2 siRNA	suppression of Bcl2 (a key regulator among the anti-apoptotic proteins) resulted in DOX enhanced antitumor efficacy	[101]
complex polymer micelles (P85-PEI/TPGS^e^)	paclitaxel (PTX) + survivin shRNA (shSur)	down-regulation of survivin, enhanced paclitaxel-induced apoptosis and cell arrest in the G2/M phase	[102]
micelleplexes (pH-responsive cationic micellar nanoparticles; PDMA-*b*-PDPA^f^)	paclitaxel (PTX) + Bcl-2 siRNA	suppression of Bcl2 (a key regulator among the anti-apoptotic proteins) resulted in enhanced antitumor efficacy of PTX	[103]
multilayered PAA cleavable pH-responsive nanoceria (FA-PAA-PNC^g^)	doxorubicin + ganetespib	ganatespib synergizes and accelerates therapeutic efficacy of DOX via ROS production	[104]
multistage solid lipid NPs (SLNs) loaded in microspheres	afatinib (AFT) + paclitaxel (PTX)	synergistic effect of afatinib and PTX due to inactivation of p70 s6 kinase by PTX and inactivation of PI3K/AKT/mTOR	[105]
liposomes (PEGylated lipo-DTX/siRNA NPs^h^)	docetaxel (DTX) + Bcl-2 siRNA	suppression of Bcl2 (a key regulator among the anti-apoptotic proteins) resulted in enhanced DTX antitumor efficacy	[106]
nanostructured lipid carriers (LHRH-decorated NLC-PTX-siRNA^i^)	paclitaxel (PTX) + gefitinib (GEF) + siRNA targeted to EGFR	i) suppression of EGF tyrosine kinase signaling pathways, ii) prevention of EGF receptor protein synthesis, and iii) induction of cell death by the microtubule-stabilizing drug paclitaxel lead to an enhanced therapeutic effect in EGFR TKI resistant cancer	[107]
polymer-coated magnetic NPs (FA-PAA-Pt(MCO)_2_(Pt) + ganetespib^j^)	platinum cyanoximate complex + ganetespib	ganetespib effectively suppressed KRAS mutated cancer cells when co-delivered with Pt-agents and prevented multidrug resistance	[108]
PEG-modified metallic NPs (IR780@INPs-CTX^k^)	cetuximab + IR780 (sonodynamic therapy)	combinatorial treatment compensates for cetuximab-resistant mutations due to the ROS-generating potential of IR780, which promotes cell apoptosis and inhibits proliferation	[109]
PEG-modified photoresponsive metallic nanocarriers	gefitinib + CuS (photodynamic switching)	re-sensitization to gefintinib as a result of the inactivation of bypass signaling in gefitinib resistant tumors due to increased ROS levels which downregulate expression of IGF1R and its downstream AKT/ERK/NF-kB signaling	[110]

^a^PLGA-PEI NPs covered with a PEI corona; ^b^methoxy poly(ethylene glycol)–poly(ʟ-lactide) NPs; ^c^poly(ethylene glycol)–poly(lactic acid) NPs; ^d^doxorubicin–polyethyleneimine conjugate via a pH-sensitive linker mixed with Bcl2 siRNA; ^e^Pluronic P85–polyethyleneimine conjugate and ᴅ-α-tocopheryl–polyethylene glycol 1000 succinate complex NPs; ^f^pH-responsive poly(2-(dimethylamino) ethyl methacrylate)-*b*-poly(2-(diisopropylamino) ethyl methacrylate) NPs; ^g^folic acid-decorated polyacrylic acid-coated pH-responsive cerium oxide NPs; ^h^PEGylated cationic liposome complex with Bcl-2 siRNA; ^i^luteinizing hormone-releasing hormone decapeptide-decorated multifunctional nanostructured carriers prepared by self-assembly of liquid/solid lipids, surfactants, and cationic lipids; ^j^folate-ligated PEGylated polyacrylic acid-coated magnetic NPs with encapsulated platinum cyanoximate complex and ganetespib; ^k^PEG-modified (distearoyl-glycero-phosphorylethanolamine-PEG-COOH) iron tetroxide core nanoparticles loaded with IR780 and decorated with cetuximab.

**Figure 2:**
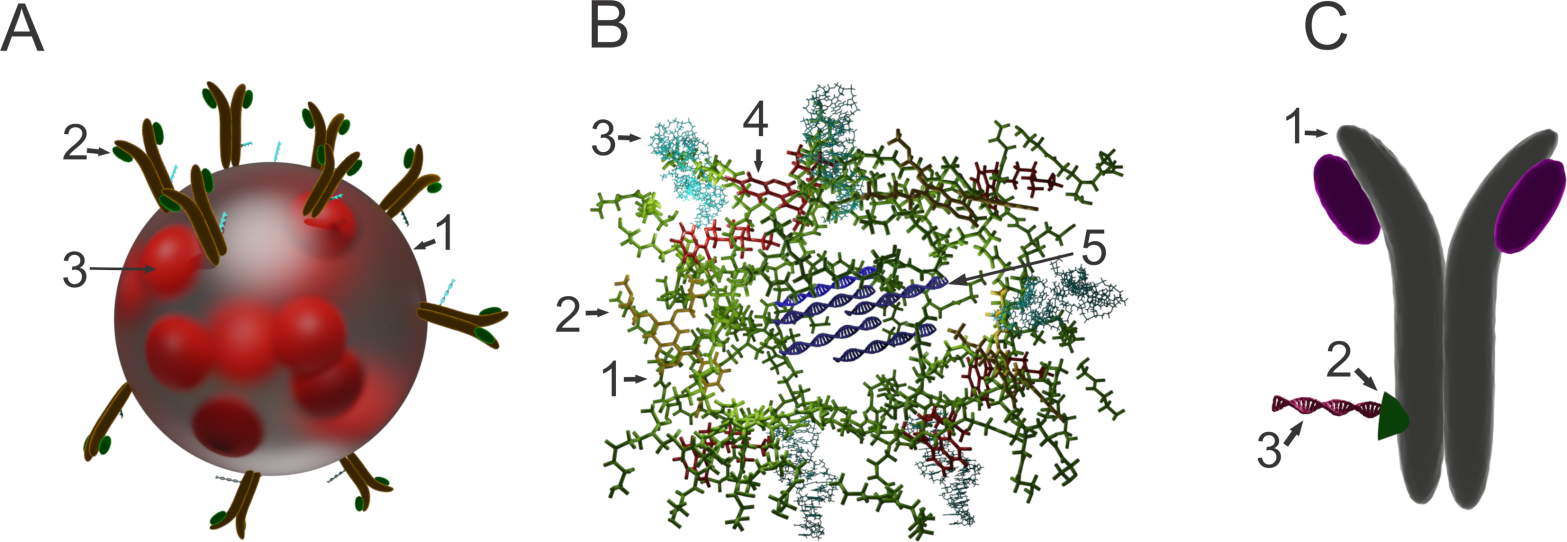
(A) Gefitinib-loaded gelatin-A NPs functionalized with a cetuximab-siRNA conjugate (Ab-SiRNA-GelGEF NPs). 1: Gelatin-A nanoparticle, 2: cetuximab–KRASG12C-specific siRNA conjugate, and 3: gefitinib [117]. (B) Multifunctional dendrimer nanocomplex. 1: poly(amidoamine) (PAMAM) nanoparticle, 2: erlotinib, 3: anti-EGFR aptamer, 4: chloroquine, and 5: survivin sh-RNA trapped in the cavity between PAMAM nanoparticles structured together in a dendrimer nanocomplex (AP/ES+CQ NPs; AP = amine-terminated PAMAM dendrimers modified with anti-EGFR aptamer; ES = erlotinib, and survivin-shRNA) [116]. (C) Cetuximab–cationic gelatin–specific siRNA delivery system (CTB-cGel-siRNA conjugate). 1: Cetuximab, 2: cationic gelatin, and 3: KRASG12C-specific siRNA [118].

**Figure 3:**
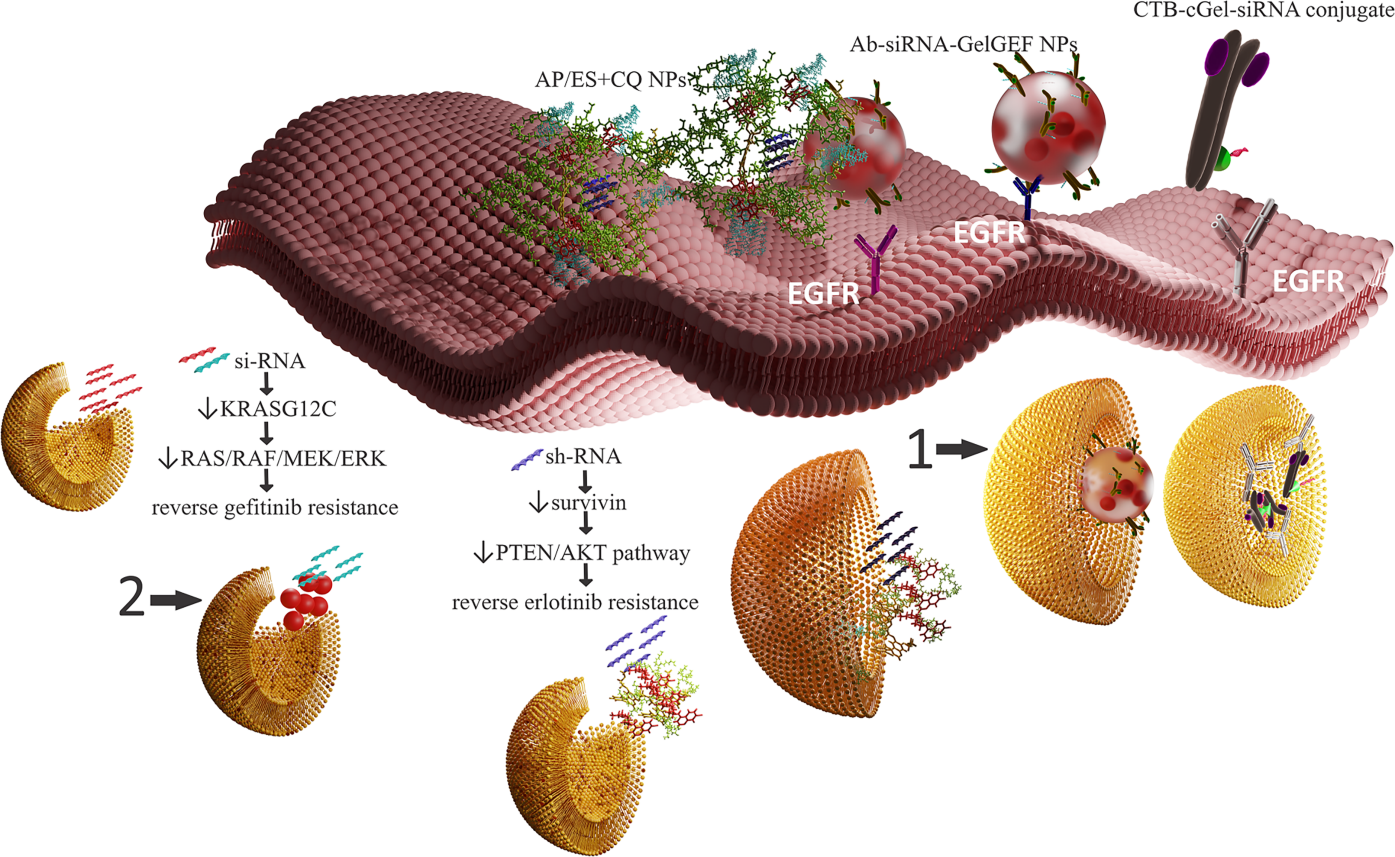
Nanotools for reversal of EGFR TKI resistance by RNAi. Favorable cell internalization was mediated by anti-EGFR aptamer/EGFR interaction (AP/ES+CQ) and cetuximab–EGFR interaction (Ab-siRNA-GelGEF NPs; CTB-cGel-siRNA conjugate). Efficient transfection was enabled by endosomal escape facilitated by (i) endosomal buffering and the proton sponge effect of chloroquine and PAMAM dendrimer (AP/ES+CQ), (ii) proton sponge effect of Ab-siRNA-GelGEF NPs, and (iii) proton sponge effect the CTB–cationic gelatin–siRNA conjugate. 1: Early endosome and 2: late endosome. This figure contains a modified version of “Liposome” by rafeequemv1 CC BY 3.0. This figure contains a modified version of "Antibodies IgG" by LucasPresoto is licensed under CC BY 4.0.

**Multistage drug delivery systems:** Multistage nanocarriers address the issue of heterogenous barriers by the use of different groups of particles carrying various functional modalities. Tasciotti et al. proposed a multistage delivery system composed of stage-1 mesoporous silica particles with improved deposition in the vascular endothelium, optimized for crossing the endothelial barrier through intravascular gaps or fenestrations or actively by a transcytotic mechanism, loaded with one or more types of stage-2 nanoparticles. The payload of drug/diagnostic agent-loaded nanoparticles optimized for improved interaction with various cancer cells, including lung cancer cells, is released over time at the tumor site, showing facilitated diffusion through tumor tissue due to their smaller size and specific surface engineering [111]. Wong et al. developed a multistage system with facilitated tumor diffusive transport composed of 100 nm gelatin nanoparticles, capable of releasing 10 nm NPs from their surface, triggered by protease degradation after tumor homing by the EPR effect [112]. Mesoporous silica vesicles (MSVs; *d*_av_ = 3 μm) with high affinity to tumor vasculature were also described by Blanco et al. as a platform for the triggered release of various therapeutic nanoscale vectors (liposomes, gold nanoshells, and microbots) and imaging contrast nanoparticles (quantum dots and iron oxide), after homing into the tumor environment [113]. Xu et al. described an injectable nanoparticle generator (iNPG) showing substantial natural tumor tropism designed as aminopropyltriethoxysilane (APTES)-functionalized nanoporous silica particles loaded with a poly(ʟ-glutamic acid) pH-cleavable linker–doxorubicin conjugate, which self-assembles into NPs after its release from the iNPG [114]. Li et al. designed a multistage nanocarrier for NSCLC targeting, composed of icotinib-loaded amphiphilic chitosan micelles with hyaluronic acid–doxorubicin NPs layered by electrostatic adsorption upon the micelle surface. Hyaluronic acid was used for CD44 targeting (a receptor that is often overexpressed on the surface of lung tumor cells), as well as for the optimization of biodistribution, improved tumor homing potential, and cell internalization of the nanocarriers. Due to the enhanced tumor accumulation, reduced accumulation at the off-site targets, and same-place/same-time delivery of therapeutic concentrations of both drugs at the site of action, an optimal synergistic effect of the active substances and efficient tumor inhibition was achieved [115]. Lv et al. prepared multifunctional dendrimer nanoscale complexes composed of anti-EGFR aptamer-modified poly(amidoamine) (PAMAM) loaded with erlotinib and chloroquine (CQ) for NSCLC treatment. These cationic nanoparticles showed high condensation capacity for survivin-small hairpin RNA (survivin-shRNA), which was trapped by electrostatic interactions in the cavity between several assembled nanoparticles (AP/ES+CQ NPs; AP = amine-terminated PAMAM dendrimers modified with anti-EGFR aptamer; ES = erlotinib and survivin-shRNA; Figure 2, Figure 3). The nanocomplexes demonstrated pH-dependent selective survivin-shRNA release in the acidic environment after endosomal escape and disassembly to single PAMAM nanoparticles showing continuous release of erlotinib and chloroquine. Chloroquine has a dual effect on the efficacy. It improves vascular barrier integrity and together with PAMAM, facilitates endosomal escape. Down-regulation of survivin reverses EGFR TKI resistance in T790M mutant NSCLC cells and sensitizes the tumor to erlotinib. The synergy of survivin and EGFR downregulation coupled with decreased angiogenesis results in significant inhibition of proliferation and improved induction of apoptosis [116].

**Biomimetic drug delivery systems:** The natural tropism of biomimetic materials for improved tissue localization has been proven to be a valuable tool in lung cancer targeting. Anselmo et al. evaluated the cell hitchhiking approach in targeting using red blood cell–polystyrene NP (200 and 500 nm) complexes (RBCsNP complexes) [119]. Compared to the free NPs, the delivery of RBCsNP complexes to the lungs, that is, the first capillary bed downstream of the IV injection of the NPs, was five-fold increased, which makes RBCsNP complexes very useful for lung tissue targeting. Once in the lung microcirculation, the RBC-bound NPs are mechanically detached from the RBCs when the RBCs are squeezed through the tiny capillaries of the air–blood barrier and transferred to the endothelium by nonspecific interactions. When decorated with vascular endothelium-specific ligands, as in the case of RBCs anti-PECAM mAb-coated polystyrene NPs, the complex showed a 760-fold increase in the lung-to-liver distribution ratio compared to nonspecific NPs. Different studies envision RBCsNPs complexes as a highly performant platform for augmented NP localization, which can be easily translated to drug delivery systems for lung and brain targeting [119–122].

Biomimetic cell membrane protein-decorated NPs successfully mitigate immune system recognition, increase blood circulation time, improve nonspecific tumor targeting, and increase tumor homing potential. NPs with red blood cell-like (RBC) surfaces, a “do not eat me” CD47 cell signal, and an immuno-suppressive protein shell instead of, or combined with, a PEG corona are among the most common biomimetic cell membrane-based NP examples in literature. So-called red blood cell vesicle shell nanoparticles (RVPNs), or RBC-mimetic NPs, showed significant retention in the systemic circulation and significantly decreased macrophage uptake compared to the conventional NPs with PEG corona [123]. Improved targeting may be achieved by RVPNs coupled with the tumor-penetrating peptide iRGD specific to αv integrins and neuropilin-1 receptors. After binding to αvβ3 and αvβ5 integrin receptors in the tumor vasculature, iRDG is subsequently cleaved by cellular proteases to a fragment with a stronger affinity for the neuropilin-1 receptor. Neuropilin-1 receptor binding triggers extravasation and initiates deep tissue penetration, intratumoral dissemination, and infiltration into the tumor parenchyma [123–125]. RVPNs decorated with a composite of an anti-EGFR single-domain antibody and iRGD provided long circulation time, improved extravasation and tumor localization, enhanced parenchymal penetration, as well as increased interaction with the overexpressed EGFR receptors [126]. Such multifunctional nanocarriers with multistage targeting hold promise for improved efficacy of treatment and increased the intracellular availability of anticancer agents for solid tumors with EGFR overexpression, among them lung cancer.

Leuko-like membrane-decorated NPs, platelet membrane-coated core–shell nanovesicles, and cancer cell membrane-coated nanoparticles are also versatile biomimetic nanocarriers showing improved biodistribution and increased tumor-homing potential [127–130]. Among them, cancer cell membrane biomimetic NPs may demonstrate specific homologous targeting to cancer cells [131]. In addition, hybrid cell membrane biomimetic shells composed of fused red blood cell membrane and homotypic cancer membrane materials may significantly contribute to personalized nanomedicine design for targeting various tumors [132].

One example of biomimetic NPs was described designed by Wang and co-workers. They used the natural tropism of mouse bone marrow mesenchymal cells (MSCs) for lung tumors for improved targeting of docetaxel (DTX) NPs (DTX-loaded polylactide-*co*-glycolide-*b*-poly(ethylene glycol); PLGA-*b*-PEG) loaded into the MSCs. The authors used animal models to show predominant lung trapping of MSCs in both rabbit and monkey. In vitro experiments in A549 NSCLC cells pointed to the release of the DTX-loaded PLGA-*b*-PEG NPs from the MSCs and their subsequent internalization. Efficient internalization and tumor inhibition were also confirmed in an in vivo lung cancer mouse model [133].

Models for drug targeting via nanocarriers and ideas for the resolution of the main drawbacks regarding their performance are numerous. Only a choice of approaches was discussed in this section giving rational solutions for improving the homing potential and decreasing off-site targets, which is one of the major issues in targeted system design. Experimental options are endless, but experience from clinical studies is still insufficient when it comes to the clinical development of nanosystems with high homing potential and acceptable toxicological profiles [134]. A systematic approach in synthesizing statistical copolymer libraries, fine-tuning nanoparticle biointeractions, and polymer bioresponsiveness, hand in hand with cell culture experiments for fast screening and dynamic cell culture models, may greatly improve the successful outcomes in the engagement of nanotools in clinical treatments in the future [135]. Recently, lipid nanocarriers gained a lot of attention owing to their ability to carry and efficiently deliver gene therapy materials and antigens. Some of the approaches for their fine-tuning for lung cancer targeting and nucleic acid co-delivery for the treatment of resistant lung cancer will be discussed below.

#### The challenge of nucleic acid tumor targeting

Silencing target genes using siRNA is an attractive therapeutic approach with significant translational potential in lung cancer treatment. The high specificity of siRNA in the downregulation of oncogenes offers numerous advantages in combinatorial lung cancer treatment for targeting mutations that contribute to the resistance to cancer therapy. Synthetic siRNA can be designed to inhibit any target gene expression and consequently prevent or decrease target protein expression, thus, altering the proliferation of cancer cells. Separate transcripts or mutations may also be specifically targeted using siRNA according to the genetic tumor profiling of resistant tumors. The increase in understanding of driver mutations of oncogenes and molecular mechanisms that contribute to cancer therapy resistance encouraged the therapeutic application of RNA interference as a powerful tool to fight resistant tumors. Knockdown of oncogenic genes involved in drug resistance combined with traditional therapy or molecularly targeted agents for subsequent tumor killing may alleviate the issue of resistance. Some of the recent approaches in the design of nanocarriers for overcoming the challenges of siRNA delivery and therapeutic nanosystems for combined RNAi treatments of resistant NSCLC will be discussed below.

The delivery of siRNA therapy to its targets in vivo is a demanding task limited by extracellular and intracellular challenges. Combinatorial therapy further complicates the right-time, right-place co-delivery of siRNA with other active ingredients due to the differences in the physicochemical properties, delivery, and stability problems. The nanoparticle core for siRNA delivery should generally be positively charged to facilitate siRNA loading by electrostatic interactions. Frequently used traditional polymer materials as nonviral vectors for siRNA encapsulation are polyethyleneimine (PEI), cationic dendrimers, phospholipids, cationic lipids, polysaccharides such as cyclodextrin, inulin, and chitosan. These polymers are used alone or combined with amphiphilic polymers for core–shell nanoparticles such as the triblock polymer poly(ʟ-lactide)-poly(ethylene glycol)-poly(ʟ-lactide) (PLLA-PEG-PLLA) to increase the stability and decrease the immunogenicity of the nanosystems [136–137].

Lipid nanoparticles (LNPs) in clinical trials are mainly composed of ionizable cationic lipids, amphipathic phospholipids, cholesterol, diffusible PEG lipids (for transient protection), and a targeting ligand [138]. After IV administration, lung capillaries receive the entire cardiac output, but successful lung localization of nanocarriers depends upon NP interaction with the endothelial cells. Lung endothelial cells are an important target for drugs and gene delivery as they are involved in processes such as inflammation, vascular permeability, and tumor growth. Also, they play an important role in cancer development [139–140]. However, efficacy or functional delivery cannot be predicted by solely considering the biodistribution. Endothelial transcytosis and improved tropism to tumor tissue/cells, internalization rate, intracellular trafficking, and endosomal release are crucial to maximize the delivery or co-delivery of active agents to the site of action in the cell [141].

State-of-the-art LNPs for siRNA gene silencing, that is, stable antisense–lipid particles (SALPs) and stable nucleic acid–lipid particles (SNALPs), were recently developed as PEGylated lipid carriers based on ionizable lipids with pKa values between 6 and 7. Onpattro^TM^ is the first RNAi therapy used for liver-based gene silencing approved by the FDA and EC and is a SNALP transfection system based on transient PEGylation. The lipid components of these benchmark LNPs for siRNA and mRNA delivery to the liver are: (i) DLin-MC3-DMA (an ionizable cationic lipid that contains amine functions with an acid dissociation constant of ca. 6.5, neutral at physiological pH and relatively non-toxic and non-immunogenic because of a low surface charge in the physiological environment), (ii) distearoyl phosphatidylcholine (DSPC), (iii) cholesterol, and (iv) PEG_2000_-C-DMG (PEGylated myristoyl glyceride, a lipid with C_14_ acyl chains). The PEGylated lipids (PEG_2000_-C-DMG) are conjugated with short anchors to the NP lipid membrane, which allows for their redistribution from the NP surface in the surrounding environment and exposure of the ionizable lipids at the surface of the LNPs. After dissociation of the PEGylated lipids, the naked surface of the particles containing the ionizable DLin-KC2-DMA, which is neutral in a biological environment, interacts with apolipoprotein E (ApoE), enabling ApoE liver-mediated targeting. Transient PEGylation facilitates not only the localization and interaction with the target cell but also improves ion pair formation between the ionizable lipid (which will become cationic at pH 4) and the anionic endogenous endosomal phospholipids. This will enable the fast release of the payload into the cytoplasm and efficacious transfection [142–148]. Besides the efforts for liver and liver hepatocyte targeting, different research groups are working on the challenge of developing lipid nanoparticles for specific organ targeting after IV administration, including lipid nanoparticles for lung targeting or targeting relevant cell types, that is, epithelial cells, endothelial cells, immune cells of the lungs, B cells, and T cells. Data regarding the biodistribution of polymers, polymer lipids, and lipid nanoparticles indicate that the internal and external nanoparticle charges are one of the most influential factors for selective organ and tissue tropism of nanostructured LNPs [149–153]. Cheng et al. added 1,2-dioleoyl-3-(trimethylammonium) propane (DOTAP), a permanently charged quaternary amino lipid, to SNALPs used for Onpattro^TM^. They reported a shift of the protein expression profile from liver to spleen and lungs. The authors also pointed to charge-mediated changes in organ distribution, depending on the type of lipid, upon IV injection of LNPs fine-tuned for organ tropism with increasing DOTAP concentration. Components of the NPs fine-tuned with DOTAP were 5A2-SC8 (a degradable dendrimer ionizable cationic lipid with p*K*_a_ < 8), 1,2-dioleoyl-*sn*-glycero-3-phosphoethanolamine (DOPE), cholesterol, 1,2-dimyristoyl-*rac*-glycerol-methoxy(poly(ethylene glycol)) (DMG-PEG; 15/15/30/3, mol/mol), and mRNA (5A2-SC8/mRNA, 20/1, wt/wt) [154–155]. Although it is still a challenge to design SNALPs for efficient tumor cell targeting, the design of lung-, spleen- and liver-specific mRNA LNPs for selective organ targeting (SORT) is evidence that there is a light at the end of the tunnel and a solution for nucleic acid delivery problems for cancer and gene therapy. Additional findings in the field emerged with the combinatorial synthesis of SORT lipid libraries, which improved screening, selection, and optimization of ionizable SORT lipids for exclusive organ/tissue localization.

Considerable effort has also been made to understand the influence of the chemical properties (head structure, tail length, degree of unsaturation, and degree of branching) of lipidoids (i.e., above cationic lipid-like materials) and the lipidoid tail structure on biodistribution and efficacy of the NPs. It has been found that imidazole-based synthetic lipidoids preferentially target the spleen, the amide-containing lipidoids contribute to increased lung targeting, and lipidoids with ester bonds in the tail tend to deliver mRNA into the liver [156]. Rational design of LNPs by in vitro and in vivo optimization of morphology and ratio of the lipid components and their physicochemical properties (polar headgroup, linker region, and type and length of hydrophobic domain), PEG amount, PEG lipid alkyl length, as well as the physicochemical properties of the LNPs (zeta potential, p*K*_a_ value, and structure and conformation of the lipid bilayer) will result in improved targeting, localization, internalization, and endosomal escape. This will increase the potency of the NPs. The FIND project is a high-throughput approach to identify and define the rational design of LNPs for functional delivery of mRNA to the liver and non-liver tissues and targeted gene editing. Structure–activity relationships of the LNPs are discovered by building and evaluating different LNP libraries [153]. Studies should also intensify in the area of nanoscale biointerface interactions and their influence on specific targeting and internalization, improved tolerability, and the reduction of immunogenicity and off-target effects that may generate systemic cytokines, activate complements, and intensify the frequency of the side effects.

A multifunctional envelope-type nanodevice (MEND), inspired by the influenza virus, for lung endothelial cell siRNA targeting was recently reported in the literature. The authors discussed that the developed Glu-Ala-Leu-Ala peptid (GALA)-MEND NPs could be clearly distinguished from conventional cationic lipoplexes and polyplexes, which are known for their high frequency and magnitude of coagulant, inflammatory, and hemolytic side effects. Conventional cationic complexes may form large aggregates with erythrocytes, which will drive lung accumulation, induce microinfarctions and ischemia and cause possible myocardial damage. The GALA-MEND structure consists of a complex of siRNA and PEI, encapsulated in a cationic liposomal envelope (di-octadecenyl-trimethylammoniumpropane (DOTMA)/egg phosphatidylcholine (EPC)/cholesterol), the surface of which was modified with cholesteryl GALA (Chol-GALA) and stearylpolyethylene glycol 2000 (STR-mPEG2000). GALA is a synthetic pH-sensitive peptide inspired by the envelope-type influenza virus. GALA improves siRNA delivery to endothelial cells after its structural transformation in the acidic environment of the endosomes, which facilitates endosomal membrane fusion and endosomal escape. This artificial virus-like vector and nucleic acid carrier showed high efficacy in targeting the lung endothelium because of lectin receptor recognition by the GALA protein. When intravenously administered to mice, due to the high targeting potential and efficacious siRNA delivery, GALA-MEND induced more than 80% gene knockdown compared to the non-treated group and successfully suppressed lung metastasis by approximately 50% compared with the control groups [157–158]. Santiwarangkool et al. tried to improve the lung-targeting potential of GALA peptide-decorated liposomes by adding a polyethylene glycol linker between GALA and the lipid surface. Liposomes modified with GALA/PEG2000 showed increased lung accumulation after IV administration in mice and were internalized more efficiently by human lung endothelial cells (HMVEC-L) compared with GALA/Chol-modified liposomes [159]. Targeting and functional efficacy of siRNA-loaded GALA/PEG2000-MENDssPalmE NPs modified with an intracellular environment-responsive lipid-like material (i.e., pH-activated ssPalmE) resulted in highly efficient knockdown of a lung endothelium specific gene in a mice model. Compared to GALA-modified NPs, GALA/PEG2000 as a ligand resulted in a more efficient gene knockdown. Abd Elwakil et al. further investigated the possibilities for improvement of gene silencing efficacy and targeting potential of GALA-MEND for siRNA delivery to the lung endothelium. They replaced DOTMA with a pH-sensitive lipid (YSK05) in the LNPs. The incorporation induced a dramatic improvement in silencing efficiency by enhancing endosomal escape. However, this also caused a reduction in lung selectivity, raising the awareness that not only the targeting ligand but also the composition of the nanosystem itself heavily influences the nanoscale biointeractions and the organ selectivity. GALA/YSK05‐MEND NPs were more efficient than a previously developed MEND with a robust lung endothelium gene knockdown at small doses of 0.01 mg siRNA·kg^−1^ [160]. Hagino et al. optimized GALA/YSK05‐MEND-modified LNPs for pDNA delivery to the lungs. They prepared a double-coated MEND composed of DOPE/STR-R8 (9.55:0.45) as the inner coating, while the outer coating was composed of DOTMA/YSK05/Chol/DMG-PEG/CholGALA (4:4:2:0.3:0.4). The MEND showed a higher lung/liver ratio and efficient gene expression in the lung [161].

#### Co-delivery of cytotoxic agents, chemical inhibitors, and nucleic acids using nanocarriers

Garbuzenko et al. designed LHRH decapeptide-decorated multifunctional nanostructured lipid carriers (NLCs) for co-delivery of siRNA and paclitaxel as combined therapy for resistant NSCLC. Suppression of four types of EGFR tyrosine kinases (TKs) by a pool of siRNAs resulted in a three- to seven-fold greater in vitro efficacy of the LHRH-NLC-siRNAs-PTX combined therapy compared to gefitinib in three cell lines with decreasing order of gefitinib sensitivities, H3255, A549, and H1781, regardless of the EGFR TK mutation status. LHRH peptide may influence biodistribution and cell internalization as it is overexpressed in many types of cancer cells, including human lung cancer cells. In contrast, no detectable levels can be found in liver, kidney, spleen, heart, muscle, and lung. The LHRH-decorated nanomedicines showed a favorable lung distribution after IV and pulmonary administration in a mouse model and superior anticancer effect in a human lung orthotopic A549 mouse model compared to the treatment with a local inhalation with gefitinib [162]. Another example of LHRH-decorated multifunctional nanostructured lipid nanocarriers for EGFR-resistant cancer is described in Table 2.

Aiming to improve tumor response and regression, Xue et al. proposed lipid/polymer nanocarriers composed of 7C1 (i.e., PEI-C_15_ carbon lipid) mixed with C_14_PEG2000 for concurrent delivery of miR-34a and siKRAS, to restore the p53-regulated tumor suppressor mRNA levels in lung tumor and reduce KRAS gene expression. Restored miR-34a levels accompanied with KRAS gene knockdown and reduced MAPK signaling in KRAS-mutant cancers, increased apoptosis, and reduced tumor growth in a physiologically relevant mouse model of human lung adenocarcinoma. Multigene therapy proved to be more efficient compared to single-RNA treatment and, when combined with chemotherapy (cisplatin), further improved survival. Considering novel achievements in the field of design of LNPs with specific organ/tissue/cell tropism, combined siRNA/microRNA treatments may be used for personalized therapies targeting primary and metastatic cancer sites [163].

Kim et al. demonstrated that single or combined (Vim and/or JAK3) siRNA delivery to EGFR-overexpressing tumor cells using anti-EGFR immunolipoplexes coupled with cetuximab or immunoviroplexes decorated with fusogenic viral envelope proteins resulted in efficient reduction of cell viability and a potent anticancer effect [164].

Li et al. designed self-assembled lipid prodrugs for the delivery of therapeutic concentrations of two chemotherapeutic agents at the same time and the same place in order to avoid differences in pharmacokinetic profiles and optimize combination synergy in the treatment of NSCLC. The study highlighted the superiority of the co-delivery of PUFAylated (polyunsaturated fatty acid) cisplatin and SN38 chemotherapeutic agents assembled in PEGylated lipid nanomedicines using 1,2-distearoyl-*sn*-glycero-3-phosphoethanolamine-*N*-[methoxy-(polyethyleneglycol)2000] (DSPE-PEG2k), in comparison to single-drug therapy. The remarkable increase in the efficacy in mice harboring a cisplatin-resistant lung tumor xenograft was due to the SN38-altered DNA repair combined with the inhibition of ATM/Chk2/p53-mediated pathway, which imposes additional DNA damage. Therefore SN38, when acting at the same time as cisplatin, augments its efficacy and prevents cisplatin resistance [165].

Polymer–lipid hybrid systems or polymer systems are also described as carriers for the co-delivery of siRNA. Gao et al. suggested a combined immunotherapeutic approach involving PD-L1-siRNA and IL-2 pDNA delivered to NSCLC using PEI lipid nanoparticles (PEI-stearic acid/dipalmitoyl phosphatidylcholine/cholesterol NPs) [166]. The co-delivery of IL-2, a key regulator of T-cell activation, with PD-L1 siRNA for immune checkpoint blockade, contributes to the appropriate immune cell equilibrium and optimizes long-term anti-PD-L1/IL-2 immunotherapy [166–167]. The authors argue that the combination of PD-L1 knockdown siRNA and immunostimulatory pDNA/IL-2 delivered using LNPs reduces the drug resistance rate and leads to enhanced anti-tumor activity while also providing tumor-selective therapeutic properties [166].

Ultrasmall, less than 30 nm, theranostic micelles with a magnetic core surrounded by a DSPE-PEG2000 phospholipid layer, loaded with erlotinib and decorated with bevacizumab (Bev + Erl@MNPs) were developed by Wang et al. for the treatment of refractory NSCLCs expressing EGFR wild-type (EGFR-wt) genes. Their aim was to evaluate the capacity of the actively targeted erlotinib-based nanoscale agent to sensitize wild-type EGFR to TKIs. The authors pointed to a dual effect involving the successful sensitization of EGFR-wt to erlotinib, potent tumor inhibition, and bevacizumab-induced normalization of the tumor-embedded vessels in a mouse model. Vascular normalization as an additional effect to the co-regulatory relationship and dual inhibition of VEGF and EGFR pathways was proven to be a promising strategy to enhance cell apoptosis of NSCLC cells in vivo [168]. Such combination therapies, including the co-delivery of cetuximab and afatinib, are useful in restoring the sensitivity towards third-generation specific mutation inhibitors in tumors with acquired resistance, including Src-AKT pathway activation and the recently reported EGFR wild-type allele amplification [169].

Recently, two cetuximab-decorated gelatin-based KRASG_12_C-specific siRNA delivery systems showing successful KRAS oncogene knockdown leading to sensitization of the cancer cells to gefitinib were described in the literature. In the cetuximab–cationic gelatin–KRASG12C-specific siRNA gelatin antibody delivery system (GADS; CTB-cGel-siRNA conjugate), positively charged gelatin had a dual role. It acted as a linker between the antibody and the siRNA and as an endosomal escape agent for efficient delivery of the siRNA in the cell cytoplasm due to its charge reversal properties (Figure 2, Figure 3) [118]. The second system composed of gefitinib-loaded gelatin-A NPs functionalized with a cetuximab-siRNA conjugate (Ab-siRNA-GelGEF NPs) also efficiently delivered stable siRNA to the cell cytoplasm in vitro and in vivo in a mouse model (Figure 2, Figure 3). This successfully sensitized the cancer cells to gefitinib through GAB1-SHP2 dissociation, disabling the feedback loop between Ras and AKT pathways and causing 70% loss in cell viability of KRAS-mutant NCI-H23 NSCLC cancer cells [117].

## Conclusion

A rigorous understanding of the challenges regarding efficient cancer cell targeting and the engineering of corresponding nanosystems is relevant to attack multiple molecular targets simultaneously, overcoming acquired drug resistance, and increasing the therapeutic potential of molecularly targeted agents, anticancer drugs and immunotherapy. Experimental options for nanomedicines with improved targeting and homing potential are endless. However, clinical studies have often resulted in dubious results and are often accompanied by an unacceptable toxicological profile. The recent focus on large-scale screening and optimization of tumor homing agent libraries for the fine-tuning of polymer NPs and the synthesis of statistical libraries of lipid NPs with balanced biointeractions and bioresponsiveness may significantly improve the outcomes of clinical treatments based on actively targeted nanotools with improved cell internalization and selective intracellular delivery. Multifunctional and multistage nanocarriers designed to overcome different barriers, to interact with the environment, and to respond to the inherent heterogeneity of the tumors should yield advantages over conventional nanocarriers. Novel strategies for precise delivery to specific intracellular targets may resolve cancer resistance issues. They may also increase the efficacy of co-delivery of combined therapy to block or knockout oncogenic genes using RNAi, reverse drug resistance, and subsequently kill the tumor using molecularly targeted or anticancer agents. Knockout of overexpressed resistance genes or removal of the functional regions of drug resistance genes to reverse the resistance of NSCLC can be performed by gene editing and CRISPR/Cas9 technology. However, additional conclusive data to ensure the feasibility of the approach are still needed.

## Appendix

Table 3 shows all abbreviations and their meanings/explantions used in the text.

**Table 3:** List of abbreviations.

Abbreviation	Meaning/explanation

Ab-siRNA-GelGEF	gefitinib loaded gelatin-A NPs functionalized with a cetuximab-siRNA conjugate
ADH	adipic acid hydralazide
AF	afatinib
AKT	protein kinase B
ALK	anaplastic lymphoma kinase
AP	amine-terminated PAMAM dendrimers modified with anti-EGFR aptamer
AP/ES+CQ	anti-EGFR aptamer modified poly(amidoamine) (PAMAM) nano complexes loaded with erlotinib/chloroquine and electrostatic interaction trapped survivin-shRNA
ApoE	apolipoprotein E
APTES	aminopropyltriethoxysilane
ATS	American Thoracic Society
Bev + Erl@MNPs	theranostic micelles with a magnetic core surrounded by a DSPE-PEG2000 phospholipid layer, loaded with erlotinib and decorated with bevacizumab
BRAF	v-raf murine sarcoma viral oncogene homolog B1
CD8	cluster of differentiation 8
CD13	aminopeptidase N
CD44	lymphocyte homing receptor
CD47	integrin-associated protein
CD274	programmed death ligand-1
Chol-GALA	cholesteryl GALA
cMET	mesenchymal-epidermal transition factor
c-MYC	cellular myelocytomatosis protein
CQ	chloroquine
CRKL	CRK like proto-oncogene, adaptor protein
CTB-cGel-siRNA conjugate	cetuximab-cationic gelatin-specific siRNA delivery system
DAC	dacomitinib
DLin-KC2-DMA	2,2-dilinoleyl-4-dimethylaminoethyl-[1,3]-dioxolane
DLin-MC3-DMA	(6*Z*,9*Z*,28*Z*,31*Z*)-heptatriaconta-6,9,28,31-tetraen-19-yl 4-(dimethylamino)butanoate
DOPE	1,2-dioleoyl-*sn*-glycero-3-phosphoethanolamine
DOTAP	1,2-dioleoyl-3-(trimethylammonium)propane
DOTMA	dioctadecenyl-trimethylammoniumpropane
DSPC	distearoyl phosphatidylcholine
DSPE	1,2-distearoyl-*sn*-glycero-3-phosphoethanolamine
DSPE-PEG2000	1,2-distearoyl-*sn*-glycero-3-phosphoethanolamine-*N*-[amino(polyethylene glycol)-2000]
DTX	docetaxel
ECT	3-mercaptopropionic acid
EGFR	epidermal growth factor receptor
EGT	thiogalactose residues
EML4-ALK -ALK	echinoderm microtubule-associated protein like 4 and anaplastic lymphoma kinase
EMT	epithelial-mesenchymal transition
ENT	cysteamine
EPC	egg phosphatidylcholine
EPR	enhanced permeability and retention
ERBB2	receptor tyrosine-protein kinase erbB-2
ERBB3	receptor tyrosine-protein kinase erbB-3
ERK	extracellular signal-regulated kinase
ERL	erlotinib
ERS	European Respiratory Society
ES	erlotinib and Survivin-shRNA
FLT3	fms-related receptor tyrosine kinase 3
GADS	gelatin antibody delivery system
GALA	peptide with glutamic acid-alanine-leucine-alanine repeats
GDP	guanosine diphosphate
GEF	gefitinib
GTP	guanosine triphosphate
HA	hyaluronic acid
HER2	human epidermal growth factor receptor 2
HGF	hepatocyte growth factor
HIF-1α	hypoxia inducible factor 1α
HMVEC-L	human lung endothelial cells
IASLC	International Association for the Study of Lung Cancer
iNPG	injectable nanoparticle generator
iRGD	red blood cell vesicle shell nanoparticles coupled with tumor penetrating peptide
JAK3	Janus kinase 3
KRAS	Kirsten rat sarcoma viral oncogene homologue
LHRH	luteinizing hormone-releasing hormone
LNP	lipid nanoparticles
LPH NPs	lipid polymer hybrid nanoparticles
MAPK	mitogen activated protein kinase
MAP2K1	mitogen-activated protein kinase 1
MEK	mitogen activated protein kinase kinase
MEND	multifunctional envelope-type nano device
MET	mesenchymal-epitehelial transition factor
MPS	mononuclear phagocytic system
mRNA	messenger RNA
MSC	mouse bone marrow mesenchymal cells
MSVs	mesoporous silica vesicles
mTOR	mammalian target of rapamycin
NF-1	neurofibromin 1
NLC	nanostructured lipid carriers
NPs	nanoparticles
NSCLC	non-small cell lung cancer
OS	osimertinib
PAGE	poly(allyl glycidyl ether)
PAMAM	poly(amidoamine)
PCL	polycaprolactone
PD-1	programmed cell death protein 1
PD-L1	programmed death-ligand 1
PDMAEMA	poly(2-dimethylamino)ethyl methacrylate
pDNA	plasmid DNA
PECAM	platelet/endothelial cell adhesion molecule 1
PEG	poly(ethylene glycol)
PEG2000-C-DMG	PEGylated myristoyl glyceride, lipid with relatively short C14 acyl chains
PEI	polyethyleneimine
PIK3CA	phosphatidylinositol 3 kinase, catalytic subunit alpha
PI3K	phosphoinositide 3-kinase
PLGA	poly lactic-*co*-glycolic acid
PLLA	poly(ʟ-lactide)
PtBGE	poly(*tert*-butyl glycidyl ether)
PTEN	phosphatase and tensin homologue
PTX	paclitaxel
PUFA	polyunsaturated fatty acid
RAF	rapidly accelerated fibrosarcoma
RBC	red blood cells
RBCsNPs complexes	red blood cell nanoparticle complexes
RET	rearranged during transfection proto-oncogene gene
ROS1	proto-oncogen tyrosine protein kinase
RTKs	receptor tyrosine kinases
RVPNs	red blood cell vesicle shell nanoparticles
SALPs	stable antisense–lipid particles
SCLC	small cell lung cancer
shRNA	short hairpin RNA
siRNA	short interfering RNA
SNALPs	stable nucleic acid–lipid particles
SORT	selective organ targeting
ssPalmE	vitamin E scaffolded SS-cleavable and pH-activated lipid-like material
SSTRs	somatostatin receptors
STR-mPEG2000	stearylpolyethylene glycol 2000
TKIs	tyrosine kinase inhibitors
TPP	triphenylphosphonium
VEGFR	vascular endothelial growth factor receptor
YSK05	1-methyl-4,4-bis[(9*Z*,12*Z*)-9,12-octadecadien-1-yloxy]piperidine
